# Label-Free Detection of Single-Base Mismatches in DNA by Surface-Enhanced Raman Spectroscopy**

**DOI:** 10.1002/anie.201102776

**Published:** 2011-07-19

**Authors:** Evanthia Papadopoulou, Steven E J Bell

**Affiliations:** Innovative Molecular Materials Group, School of Chemistry & Chemical Engineering, Queen's UniversityBelfast BT9 5AG (UK); * Innovative Molecular Materials Group, School of Chemistry & Chemical Engineering, Queen's UniversityBelfast BT9 5AG (UK)

**Keywords:** DNA, nanomaterials, nucleotides, RNA, SERS

Detection of specific DNA sequences and identification of single-nucleotide polymorphisms (SNPs) are both important for diagnostic purposes, since alterations in the DNA sequence are the cause of the most common inherited disorders.^1^ Methods for label-free identification of short DNA sequences are currently relatively limited. The most obvious choice is MALDI-TOF mass spectrometry, which is extensively used in SNP genotyping methods and has high sensitivity.^2^ However, MALDI-TOF equipment is expensive, which partly offsets the significant advantage of being able to avoid labeling steps in the analysis. Surface-enhanced Raman spectroscopy (SERS) is an obvious alternative method, which has the potential to combine high sensitivity with low cost.^3^ However, SERS studies of DNA most often involve detection of SERS labels;^4^ even those that do not are typically carried out on sequences that have been thiolated to promote surface binding.^5^ This approach not only increases the cost of the assay, but also the specific binding through a sulfur group gives the DNA a more tilted orientation that may prevent all the bases from interacting sufficiently with the substrate to provide good Raman signals. Indeed, it has been reported that the SERS spectra of thiolated DNA are completely dominated by adenine signals.^5^ A recent approach utilized TERS (tip-enhanced Raman spectroscopy)to record the spectra of an unthiolated single poly C strand, in order to work towards a method of obtaining sequence information by moving the TERS probe in intervals of one base-to-base distance.^6^ This approach, although promising, requires complex instrumentation, and, additionally, acquisition of Raman signals with single-base sensitivity is extremely challenging.

Herein we show that it is possible to obtain distinctive SERS spectra of unthiolated DNA sequences, which can be used to detect a single-nucleotide mismatch. This detection can be achieved by adsorbing the sequences nonspecifically through the nucleotide side chains onto the enhancing surface of silver colloids, where the sequences adopt a flattened configuration. To this end, the colloids were aggregated with an electrolyte that does not bind strongly in the silver surface (MgSO_4_ in this case),^7^ and therefore allowed the DNA sequence to bind through its constituent bases. Previously, we demonstrated that silver colloids can provide very large SERS enhancements for the negatively charged mononucleotides when colloids were aggregated with MgSO_4_.^7^ We applied the same principle for the detection of single-base mismatches in short DNA sequences.

Initially, parallel studies were carried out using both thiolated and unthiolated 30-mer DNA-1 sequences (Table 1), which were either probed directly or after thermal pretreatment, as previously described.^5^ All the samples gave acceptable SERS signals, as shown in Figure 1, thus demonstrating that thiolation is not necessary to obtain high-quality SERS spectra of DNA. In fact the unthiolated samples gave signals that were at least as large as the thiolated analogues. The spectra of the thermally treated thiolated sample strongly resemble the spectra of adenine,^8^ which agrees with previous data obtained on Au substrates and was attributed to adenine having a stronger scattering cross-section than the other bases.^5^ Interestingly, the SERS spectra of the unthiolated and thiolated form with no thermal pretreatment are different from the thermally treated sample, but similar to each other. This result suggests that the untreated thiolated sample adopts a similar adsorption configuration to the unthiolated sample, which is believed to lie flat with respect to the surface because of strong nonspecific binding between the bases and the metal surface. Similar binding interactions presumably also occur with thiolated strands^9^ if they are not heat-treated. However, the most important point is that the spectra of simple unthiolated sequences are not dominated by the adenine modes, but show bands that arise from all the bases.

**Figure 1:**
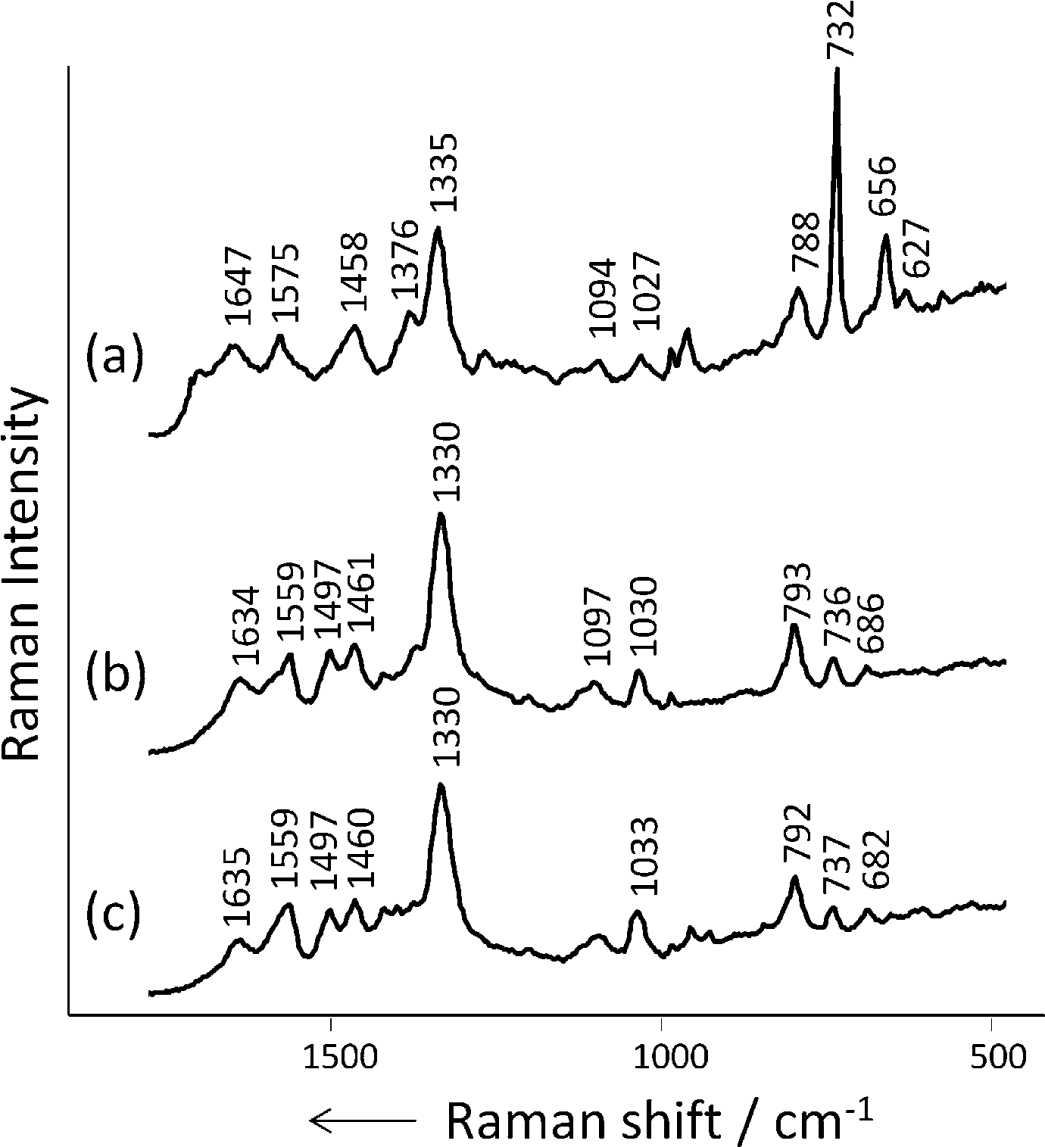
SERS spectra of 10^−5^ m solutions of a) thermally treated thiolated HS-DNA-1, b) untreated, thiolated HS-DNA-1, and c) unthiolated DNA-1. All spectra were recorded on citrate-reduced Ag colloids and aggregated with 0.1 m MgSO_4_.

**Table 1:** DNA sequences used in this study.

Code	Sequence (5′–3′)
DNA-1	GACTGCGACCAACCTAGCCTGCTATGATGT
HS-DNA-1	HS-CH_2_-DNA-1
DNA-2	CTT-TTT-CCT-GCA-TCC-TGT-CTG-GAA-*A*
DNA-3	CTT-TTT-CCT-GCA-TCC-TGT-CTG-GAA-*G*
DNA-4	ATA-AAT-CGC-CAT-TCG-TTG-ACT-A*A*
DNA-5	ATA-AAT-CGC-CAT-TCG-TTG-ACT-A*C*

To aid the analysis of the DNA spectra, the SERS spectra of the three nucleobases poly A, poly C, and poly T were recorded and used as reference spectra. The SERS spectrum of dGMP is shown instead of poly G because poly G forms secondary structures through Hoogsteen base pairing.^10^ We have found that these structures are not sufficiently flexible to allow the nucleobases to directly adsorb to the surfaces of the Ag nanoparticles, and they therefore give only weakly enhanced Raman signals. For this work, hydroxylamine-reduced Ag colloids were used as the enhancing media because they are easier to synthesize than citrate-reduced colloids, and the low concentration of Cl^−^ ions introduced during their synthesis does not affect DNA binding. Indeed the SERS spectra (Figure S1 in the Supporting Information) show that when DNA is added to colloid aggregated with MgSO4, it displaces the surface chloride layer and the 244 cm^−1^ Ag–Cl band disappears. However, if the colloid is aggregated by using a high concentration of NaCl, no DNA Raman signals are observed because the DNA cannot compete with the high concentration of NaCl (0.1 m) for surface sites.

Irrespective of these considerations, the main point is that by using suitable conditions, the SERS spectra of unthiolated homopolymers or mononucletides can be obtained (Figure 2); these spectra give characteristic bands that can be used for the identification of each nucleotide in a DNA sequence. For example, in the spectrum of the unthiolated form of DNA-1 (Figure 1 c), the bands at 737 and 682 cm^−1^ can be assigned to the ring breathing modes of poly A and poly G, respectively. The band at 792 cm^−1^ can be assigned to the ring breathing modes of poly C and poly T, which overlap. The band at 1635 cm^−1^ is assigned to the carbonyl stretching mode of poly C, while the intense 1576 cm^−1^ poly G band is observed as a shoulder of the adenine band at 1559 cm^−1^.

**Figure 2:**
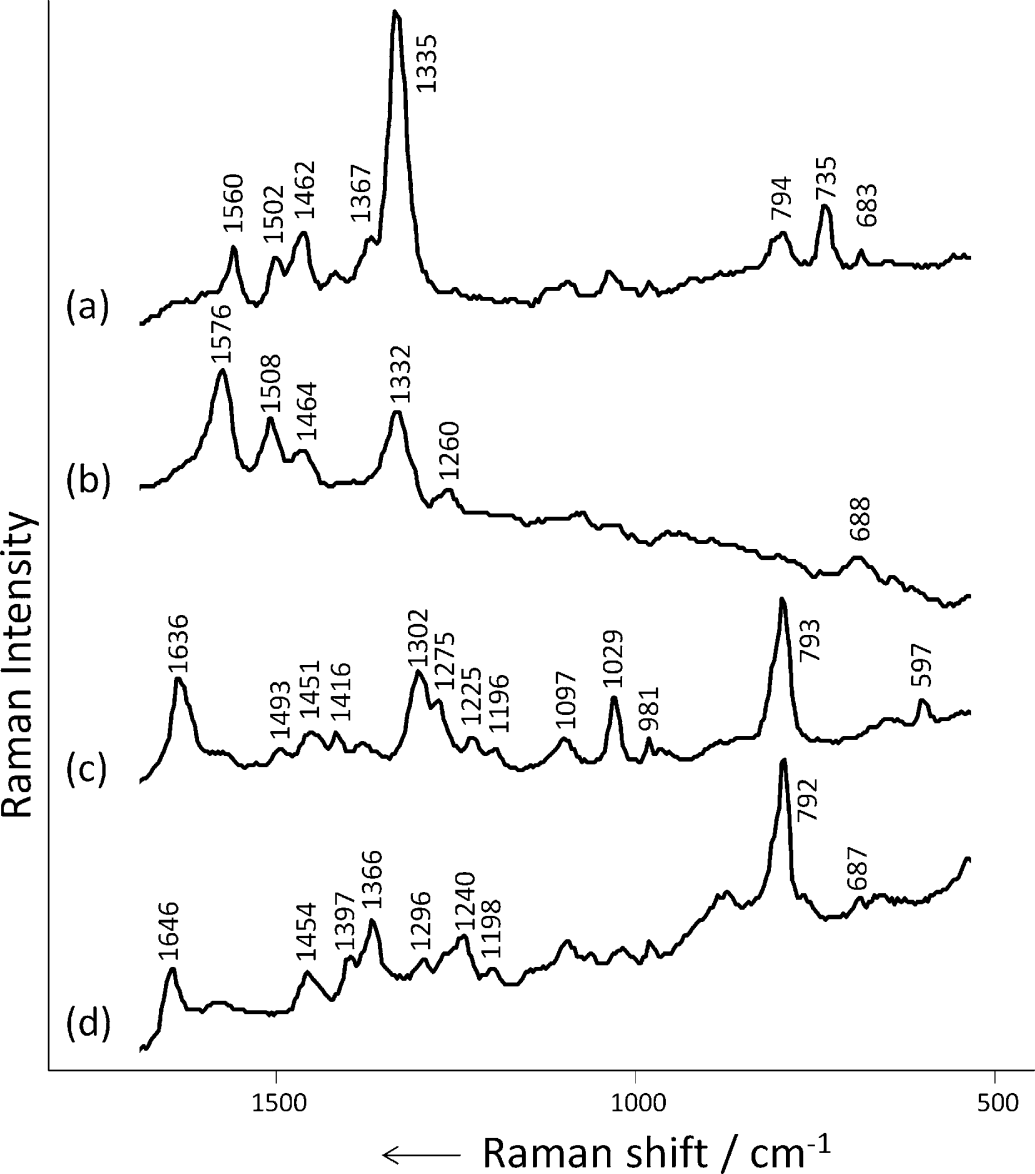
SERS spectra of 10^−6^ m solutions of the 24-mers a) poly A, b) 10^−5^ dGMP, c) poly C, and d) poly T on hydroxylamine-reduced Ag colloid aggregated with 0.1 m MgSO_4_.

Given that the SERS spectra of the unthiolated DNA strands show features associated with all the constituent bases, in principle it should be possible to detect even relatively small changes in the DNA sequence. We have tested the ability to detect A→G and C→A polymorphisms in 25-mer and 23-mer DNA sequences. Figure 3 shows the SERS spectra of DNA-2 and DNA-3, where the adenosine nucleotide in DNA-2 is replaced by a guanosine nucleotide in DNA-3. Most of the features appear unchanged but the band at 1559 cm^−1^ in the DNA-2 spectrum is broader and shifts to 1569 cm^−1^ in the DNA-3 spectrum. This observation is consistent with the strongest dGMP band, which lies at 1576 cm^−1^ and increases in intensity in DNA-3.

**Figure 3:**
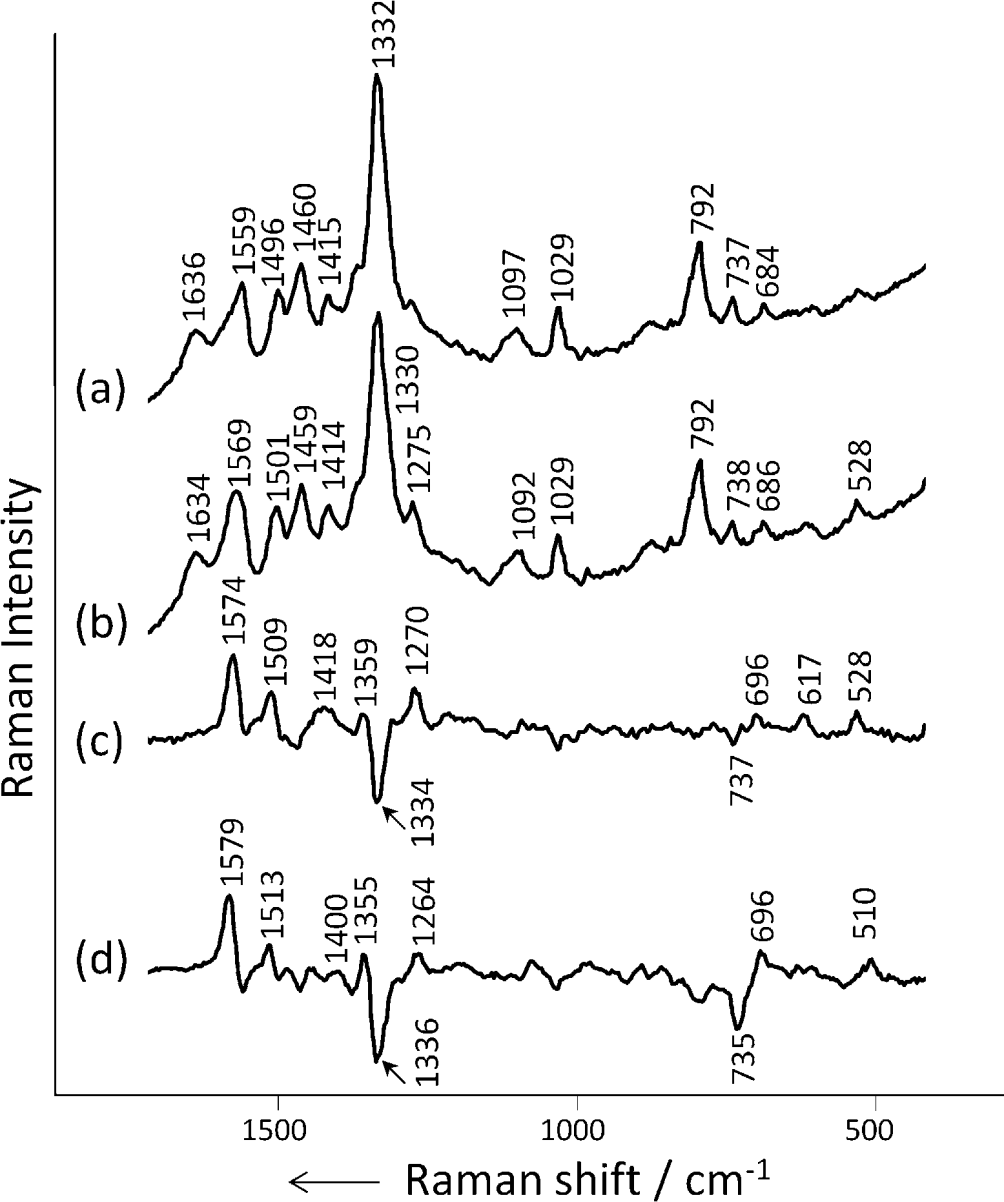
SERS spectra of a) DNA-2 and b) DNA-3. c) Simple difference spectrum of DNA-3 minus DNA-2 ((b)−(a)) shown with its intensity expanded three times. d) Model difference spectrum, dGMP minus poly A. All spectra are for hydroxylamine-reduced Ag colloids aggregated with 0.1 m MgSO_4_.

The best way to highlight the changes in the spectra is to digitally subtract them, which should remove the contributions from unchanged nucleotides to give difference spectra that contain positive and negative features corresponding to the exchanged nucleotides. Figure 3 c shows the experimental difference spectrum. Negative bands that can be assigned to poly A vibrations are observed at 1334 cm^−1^ and 737 cm^−1^. Positive G bands are observed at 1574 and 1509 cm^−1^, the expected increased band at 1332 cm^−1^ is cancelled by the stronger negative band at 1334 cm^−1^, which corresponds to adenine. More strikingly, when the experimental difference spectrum is compared to a model spectrum created by subtracting the spectra of poly A from dGMP (Figure 3 d), the agreement between the experimental and predicted pattern of band intensity changes is excellent. This result provides clear evidence that the changes in the spectra are due to the A→G polymorphism.

Each polymorphism would be expected to give a different pattern of band changes. For example, Figure 4 shows SERS data for DNA-4 and DNA-5, which correspond to C→A polymorphism. Again, small changes can be observed at the expected parts of the raw spectra, for example, the ratio of the intensity of the C band at 792 cm^−1^ to that of the A band at 737 cm^−1^ is slightly lower in the spectrum of ssDNA-5. However, the best evidence again comes from the difference spectra where the experimental data can be compared to the model obtained by subtracting the spectrum of poly C from that of poly A. Again there is excellent agreement between the expected and observed changes.

**Figure 4:**
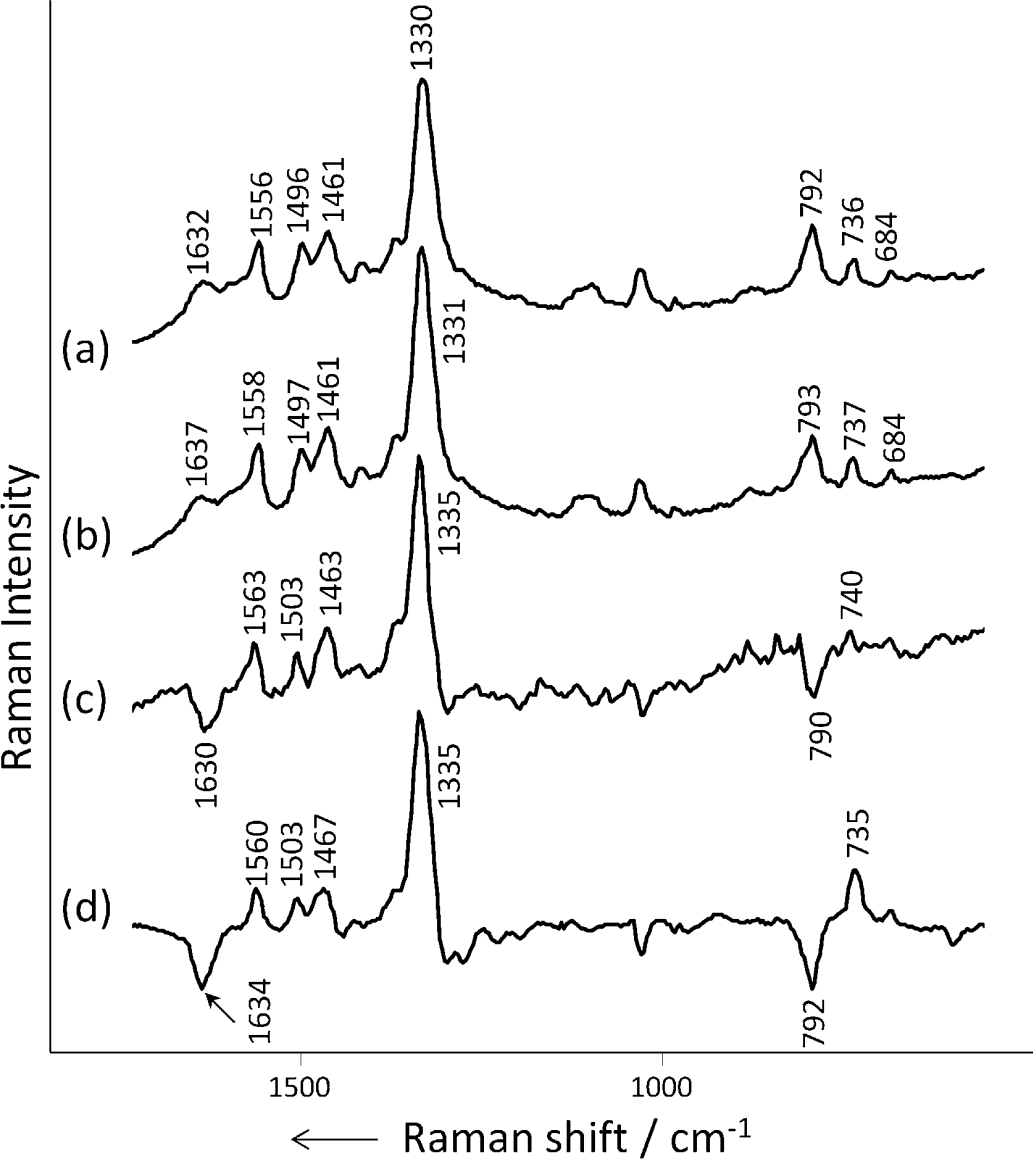
SERS spectra of a) DNA-4 and b) DNA-5. c) Simple difference spectrum of DNA-5 minus DNA-4 ((b)−(a)) shown with its intensity expanded eight times. d) Model difference spectrum, poly A minus poly C. All spectra are for hydroxylamine-reduced Ag colloids aggregated with 0.1 m MgSO_4_.

This work demonstrates that the SERS spectra of simple unthiolated DNA can be obtained under appropriate experimental conditions. These spectra show bands that are characteristic of the constituent bases and are sufficiently reproducible that differences arising from single-base mismatch can be identified in short DNA strands.

## Experimental Section

All DNA oligomers were purchased from Eurogentec Ltd. (Belgium) and underwent sePOP (selective precipitation optimized process) desalting purification by the vendor. The unthiolated DNA sequences were dissolved in doubly distilled deionized water. The thiolated DNA-1 was thermally pretreated by heating the solutions to 90 °C for 10–15 min and then rapid cooling in an ice bath. The SERS spectra were recorded on an Avalon Instrument RamanStation R1 (785 nm diode laser excitation). Typical exposure times were 2×60 s.

## Supplementary material

Detailed facts of importance to specialist readers are published as ”Supporting Information”. Such documents are peer-reviewed, but not copy-edited or typeset. They are made available as submitted by the authors.

## References

[1] Syvanen AC (2001). Nat. Rev. Genet.

[2] Tost J, Gut IG (2005). Clin. Biochem.

[3] Bell SEJ, Sirimuthu NMS (2008). Chem. Soc. Rev.

[4a] Faulds K, McKenzie F, Smith WE, Graham D (2007). Angew. Chem.

[b11] (2007). Angew. Chem. Int. Ed.

[4b] Graham D, Faulds K (2008). Chem. Soc. Rev.

[5] Barhoumi A, Zhang D, Tam F, Halas NJ (2008). J. Am. Chem. Soc.

[6] Bailo E, Deckert V (2008). Angew. Chem.

[b12] (2008). Angew. Chem. Int. Ed.

[7] Bell SEJ, Sirimuthu NMS (2006). J. Am. Chem. Soc.

[8] Papadopoulou E, Bell SEJ (2010). J. Phys. Chem. C.

[9] Storhofff JJ, Elghanian R, Mirkin CA, Letsinger RL (2002). Langmuir.

[10] Poon K, Macgregor RB (1998). Biopolymers.

