# Supplementary material for: Label-Free Detection of Single-Base Mismatches in DNA by Surface-Enhanced Raman Spectroscopy
Source: Angew Chem Int Ed Engl. 2011 Jul 19;50(39):9058–61. doi: 10.1002/anie.201102776 (PMC3625740; doi:10.1002/anie.201102776)
Supplement: Supplementary file 1 [file anie0050-9058-SD1.pdf]

Angewandte Chemie
Eine Zeitschrift der Gesellschaft Deutscher Chemiker

Supporting Information
© Wiley-VCH 2011
69451 Weinheim, Germany

Label-Free Detection of Single-Base Mismatches in DNA by Surface-Enhanced Raman Spectroscopy**

*Evanthia Papadopoulou and Steven E. J. Bell**

anie_201102776_sm_miscellaneous_information.pdf

Experimental

Silver nitrate (99.9999%), hydroxylamine hydrochloride (99.9999%), trisodium citrate, magnesium sulphate, sodium hydroxide and Tris-EDTA (TE, pH 7.4) buffer were purchased from Sigma-Aldrich. All DNA oligomers, thiolated and unthiolated used in this study were purchased from Eurogentec Ltd. (Belgium) and underwent sePOP (Selective Precipitation Optimized Process) desalting purification by the vendor. The unthiolated DNA sequences were dissolved in doubly distilled deionised (DDI, resistivity up to 18.2 MΩ cm) water from a Barnstead NANOpure Diamond™ system. The thiolated DNA was dissolved in TE buffer. Thermal pretreatment of the thiolated DNA-1 involved heating the DNA solutions to 90°C for 10-15 min and then rapid cooling in an ice bath. All glassware was washed with aqua regia followed by extensive rinsing with water prior to use.

The hydroxylamine hydrochloride reduced silver colloids were prepared according the standard procedure of Leopold and Lendl[1] (particle size ca. 80 nm). Citrate reduced silver colloids were prepared according the standard procedure of Lee and Meisel[2] (particle size ca. 113 ± 21 nm). For SERS measurements, 50 µL of colloidal solution was mixed with 50 µL of analyte solution and 25 µL of aggregating agent solution (0.1 M $MgSO_4$).

The SERS spectra were recorded on an Avalon Instrument RamanStation R1. This instrument uses a 785 nm diode laser and an echelle spectrograph. The laser power was 100 mW and spectra were typically recorded with an exposure time of 2 x 60s in 96 well polyethylene microtitre plates.

[1] N. Leopold, B. Lendl, *J. Phys. Chem. B* **2003**, *107*, 5723-5727.

[2] P. C. Lee, D. Meisel, *J. Phys. Chem.* **1982**, *86*, 3391-3395.

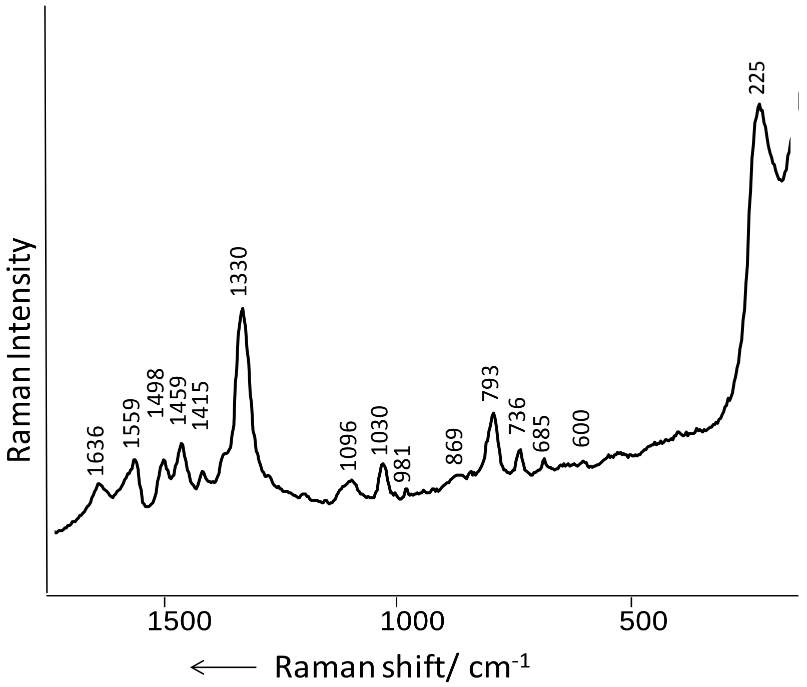

Figure S1. SERS spectrum of 10^{-5} M DNA-1 on hydroxylamine reduced Ag colloid aggregated with 0.1 M $MgSO_4$. The absence of the strong Ag-Cl band at 244 cm^{-1} shows that when DNA is added to the colloid, it displaces the surface chloride layer.

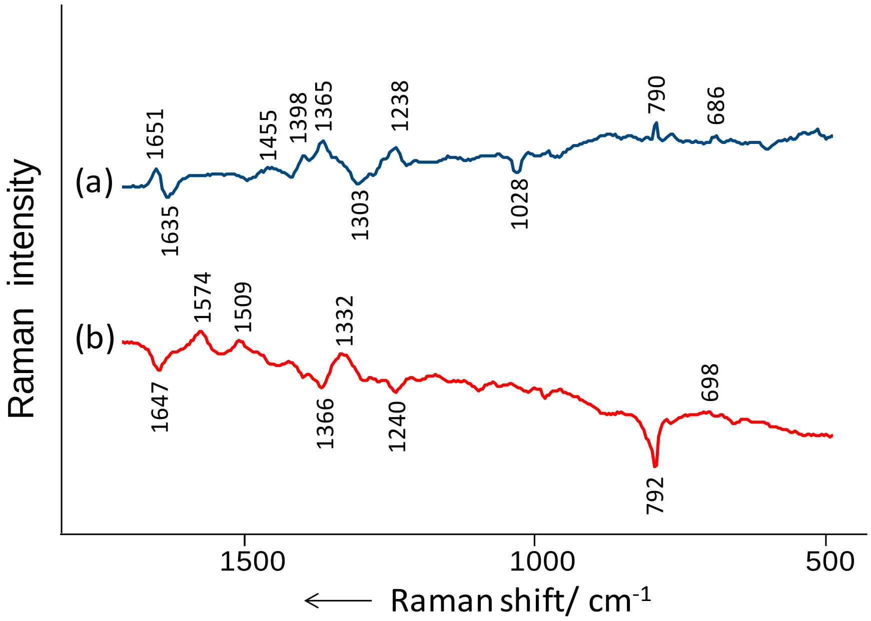

Figure S2. Model difference spectra of other possible mismatches: (a) poly T minus poly C, (b) dGMP minus poly T. All spectra used hydroxylamine reduced Ag colloid aggregated with 0.1 M $MgSO_4$.
